# The importance of intraoperative echocardiography in the early detection of mitral regurgitation as a postsurgical sequel of aortic valve replacement: a case report

**DOI:** 10.1186/s13256-023-04176-6

**Published:** 2023-11-01

**Authors:** Mehrdad Jafari Fesharaki, Tooba Akbari, Fariba Bayat, Erfan Ghadirzadeh, Elham Charkazi

**Affiliations:** 1 Fellowship of Echocardiography, Department of Cardiology, School of Medicine, Cardiovascular Research Center, Shahid Beheshti University of Medical Sciences, Tehran, Iran; 2 Cardiovascular Research Center, Mazandaran University of Medical Sciences, Sari, Iran; 3 Semnan University of Medical Sciences, Semnan, Iran

**Keywords:** Mitral regurgitation, Aortic valve replacement, Mitral leaflet perforation, Case report, Echocardiography

## Abstract

**Background:**

Mitral leaflet perforation (MLP) can rarely be a consequence of aortic valve replacement (AVR), resulting in mitral regurgitation (MR). Determining the cause and severity of MLP following AVR is crucial in preventing hemodynamic consequences, such as pulmonary hypertension and biventricular remodeling. However, the diagnosis of this rare complication requires detailed echocardiographic evaluations.

**Case presentation:**

In this paper, we report a 37-year-old Persian male with progressive dyspnea on exertion diagnosed with severe MR caused by anterior MLP following AVR and discuss the importance of intraoperative transesophageal echocardiography (TEE) in the proper and on-time diagnosis of this rare complication.

**Conclusion:**

During AVR procedure, an evaluation with TEE could be beneficial for identifying and treating such condition. Echocardiography is beneficial in providing real-time guidance during surgery, early detection of potential complications, treatment of such complications if present, and prevention of adverse outcomes.

## Background

Mitral leaflet perforation (MLP) can be a rare postsurgical sequel of aortic valve replacement (AVR), which results in the development of mitral regurgitation (MR) [1]. MLP can occur due to a variety of reasons, such as iatrogenic damage, endocarditis, or progressive deterioration of the mitral valve apparatus [2]. Iatrogenic causes include mechanical trauma to the mitral valve during surgery, while progressive deterioration can occur due to changes in the left ventricle's geometry or function [3, 4]. Additionally, in nonpost-AVR situations, autoimmune diseases such as systemic lupus erythematosus or antiphospholipid syndrome could also contribute [3]. The aortic and mitral valves are synergistically related in a way that new or worsened MR is a crucial factor to consider when undergoing surgical AVR (SAVR) [5]. Determining the cause and severity of MLP following SAVR is crucial in preventing hemodynamic consequences, such as pulmonary hypertension (PHTN) and biventricular remodeling [4]. However, the diagnosis of this rare complication requires detailed echocardiographic evaluations. Herein, we report a case of severe MR caused by anterior mitral leaflet injury following aortic valve replacement and emphasize the importance of echocardiography in the postoperative screening of this complication.

## Case presentation

A 37-year-old Persian male prisoner was admitted to a tertiary care hospital in Tehran, Iran, with progressive dyspnea (NYHA class III-IV). His dyspnea started 5 month prior to admission in NYHA class II; however, he was not examined by a professional cardiologist at the time (was neglected at prison). He was a smoker (20 pack/year) with no history of hypertension, diabetes, or substance abuse and took warfarin in prison. During this period, he mentioned neither fever nor significant weight loss.

Ten months prior to admission, he had a history of Bental surgery for type A aortic dissection in another center. At that time, the report of a preoperative transthoracic echocardiogram (TTE) showed a calcified bicuspid aortic valve, moderate AI, and mild aortic stenosis (AS), and mild MR, with normal LV size and function. Afterwards, the patient underwent SAVR with mechanical bileaflet aortic valve. The postoperative TTE reported a well seated mechanical aortic valve with normal leaflet motion and normal gradient, and mild to moderate MR. Both preoperative and postoperative TTE were reports from another medical center and unfortunately, we did not have access to any of these echocardiographic images or any other related documents. The only document the patient had was a summary sheet from his prior hospitalization. We also did not have any reports of the postoperative cardiovascular examinations.

Upon cardiovascular examination on admission to our center, a 3/6 holosystolic murmur was heard at the apex with radiation to the axilla, in addition to a 2/6 systolic murmur at left sternal border in the second intercostal space and a click of the aortic valve. Additionally, jugular venous pressure was normal and lung were clear in auscultation. In vital signs, he had a pulse rate of 83, a respiratory rate of 15, a systolic blood pressure of 110 and a diastolic blood pressure of 60, an oxygen saturation of 100%, and a body temperature of 36.9 °C on admission. The patient was well with two negative blood cultures and normal lab results (WBC = 7100/μL; Hgb = 14.9 g/dL; Plt = 215 × 10^3^/μL; Creatinine = 1 mg/dL; CRP = 0.5 mg/dL; ESR = 7 mm/h).

**Fig. 1:**
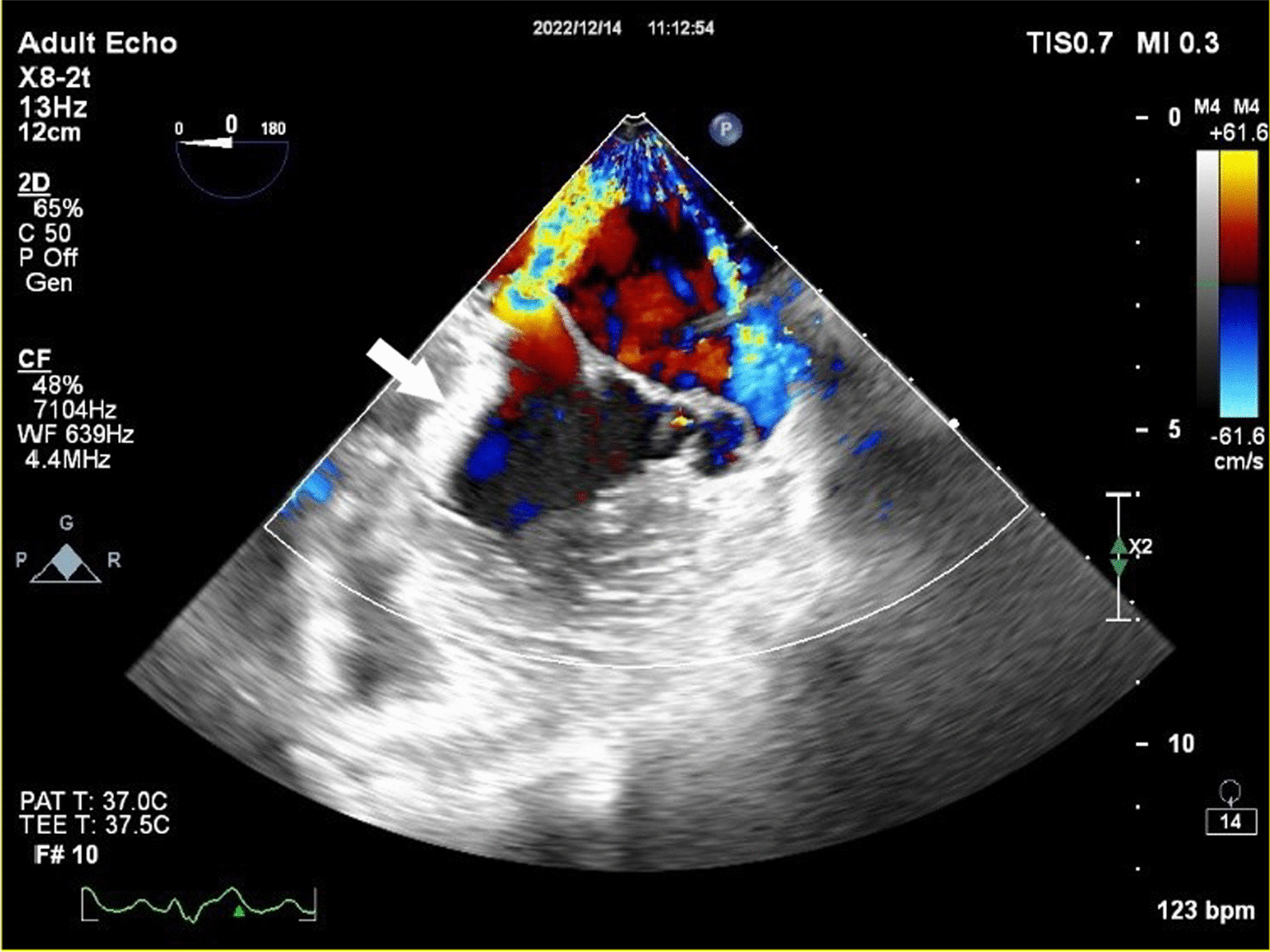
Zero degree TEE; severe regurgitation jet from the body of the AMVL; white arrow highlights mechanical aortic valve

**Fig. 2:**
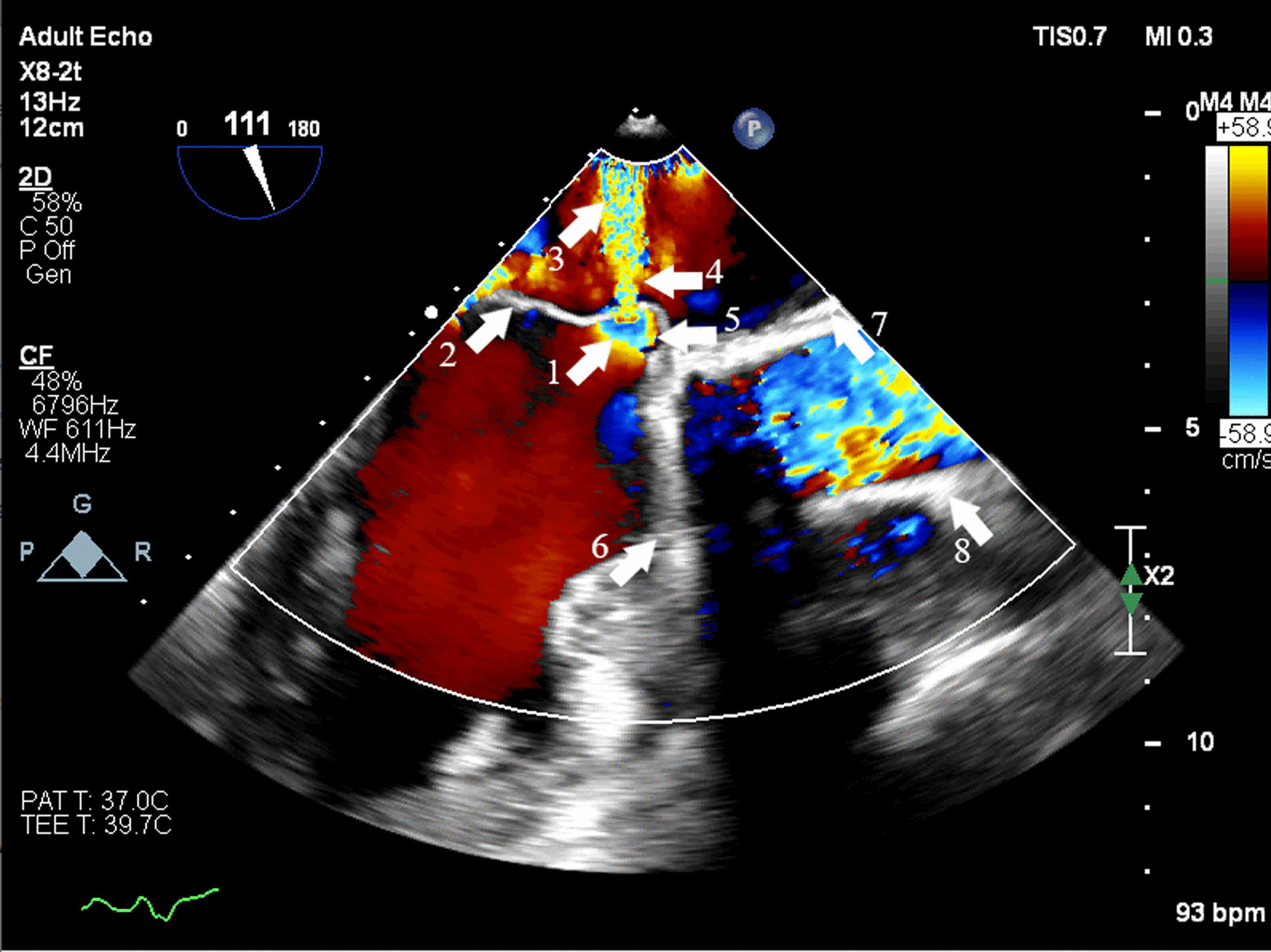
TEE long axis view; each white arrow is marked by a number which demonstrates: 1. Fenestration; 2. Anterior mitral valve leaflet; 3. Jet expansion; 4. Vena contract; 5. Flow convergence; 6. Mechanical aortic valve leaflet; 7. Ascending aortic graft; 8. Ascending aortic graft

**Fig. 3:**
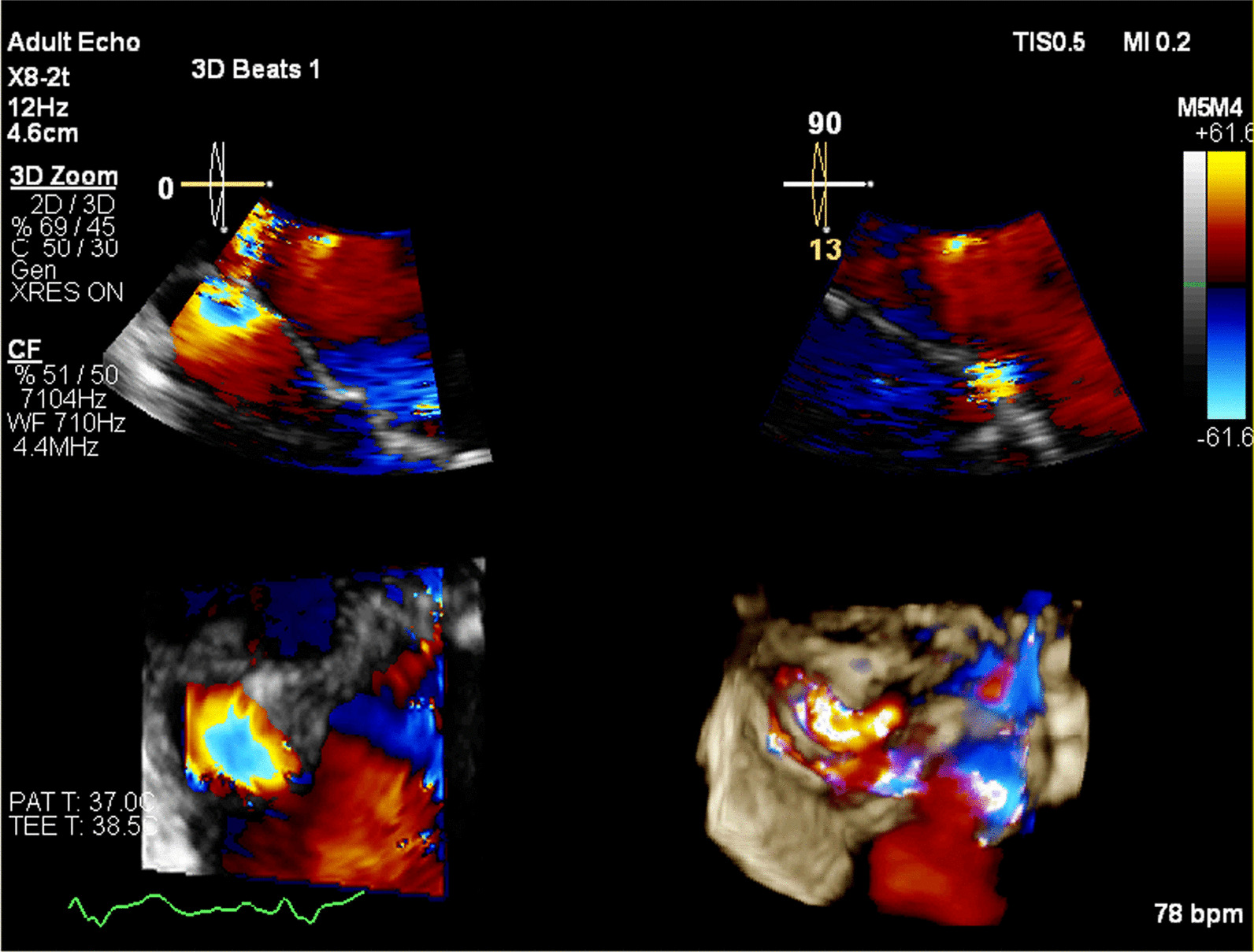
Multiplanar reconstruction from color ZOOM showed turbulency on the base of the anterior mitral valve leaflet

**Fig. 4:**
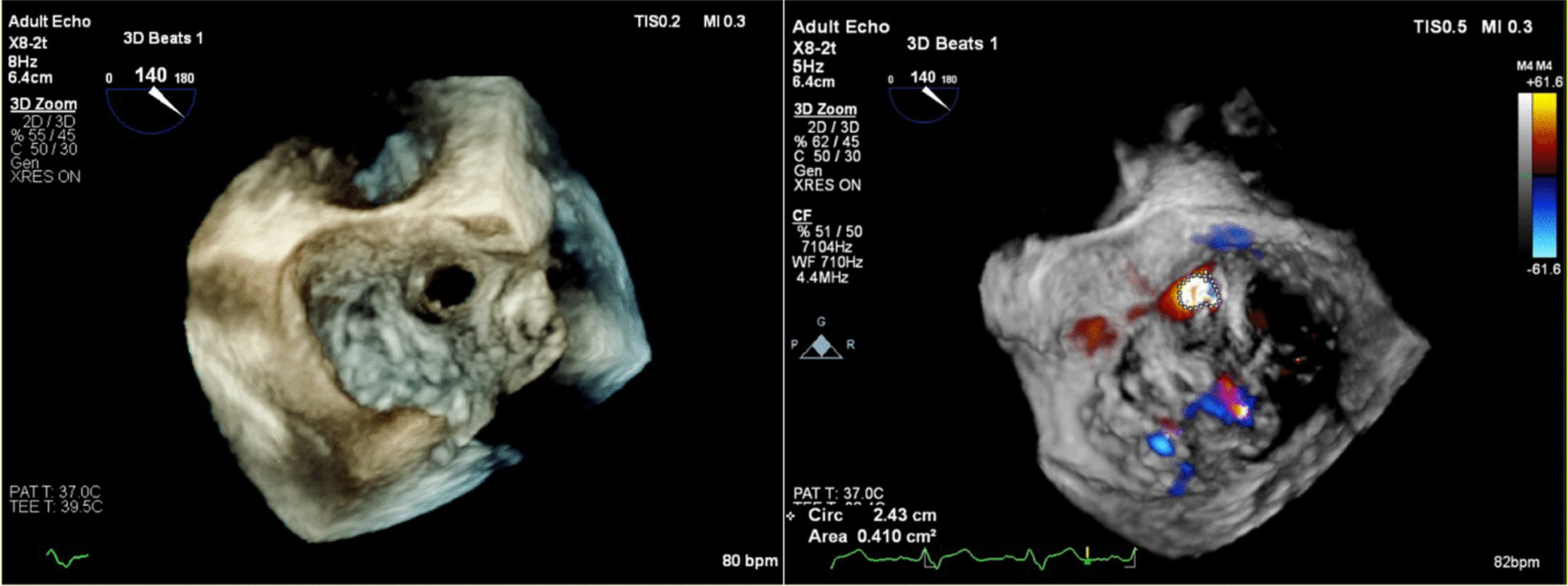
Enface 3D zoom with and without color, fenestration in A1

In the current admission, A TTE revealed severe mitral incompetence with an eccentric anterior jet**.** A transesophageal echocardiogram (TEE) showed a severe mitral regurgitation jet across the body of the anterior mitral leaflet with a vena contracta area of 0.61 cm^2^ and mild central MR (Figs. 1, 2). A 3D ZOOM image study showed a 5 × 5 mm perforation (MLP) in the anterior mitral leaflet with a crater-like appearance through the A1 segment (Fig. 3). Turbulent flow in the anterior leaflet in the region of the A1 scallop was seen in a color 3D study (Fig. 4). Other findings included moderate PHTN (estimated systolic pulmonary artery pressure of 50 mmHg), well seated mechanical bileaflet AV with normal gradient and normal leaflet motion, mild left ventricular enlargement, and significant left ventricular systolic dysfunction (LVEF: 45%). The patient underwent mitral valve replacement surgery. During open-heart surgery, direct inspection showed no signs of inflammation or infection. Finally, the patient was symptom free at all follow-up visits after the operation (last follow-up = six months after operation) with proper function and structure of cardiac valves in echocardiography.

## Discussion

The prevalence of mitral leaflet perforations is low, mainly due to infective endocarditis or autoimmune diseases. It can also be an iatrogenic complication of surgery for the aortic valve, such as forceps or needle injury [6, 7]. A review of complications in 475 cases following the repair of the aortic valve by Dyck *et al*. [8] reported two cases of perforation at the body of the anterior mitral leaflet (0.42%).

During aortic valve surgery, the anterior mitral leaflet may be damaged due to its fibrous connection to the aortic valve and close proximity to its commissure. Heavy calcification of the aortic valve and redo surgery with significant adhesions can also increase the risk of injury to the mitral valve [7]. During the surgical procedure, echocardiography can provide real-time guidance for the surgeon, ensuring the correct placement and function of the prosthetic valve [9]. It can also help identify any potential complications, perforation, or valve malposition.

Intraoperative TEE is the most essential tool for detecting damages to structures surrounding the aorta, including fenestration or tearing of the mitral valve. 3D imaging can provide a surgical perspective of the mitral valve and help predict possible complications and the successful surgery [10]. However, in our case, intraoperative TEE was not performed during his AVR procedure. TEE is the preferred imaging modality for detecting mitral valve perforation and its consequences [10]. Color Doppler jet area measurement can overestimate the severity of mitral regurgitation due to the presence of multiple MR jets and increased fluid entrainment from adjacent regurgitant jets [11–13]. A volumetric method, such as calculating total regurgitant volume and the regurgitant fraction, is more accurate. If available, 3D TEE can provide more anatomical details regarding the location and severity of mitral perforation.

Endocarditis is sometimes associated with leaflet perforation, and it is important to exclude infection in all patients with this condition. Perforations in the anterior leaflet may be the only cause of MR; if they are large, they might cause serious left ventricular failure and require early intervention. In this case, two blood cultures were negative, and no vegetation was seen on TEE. Additionally, there was no evidence from lab results, and clinical state of the patient in favor of endocarditis, so it seems that the perforation was iatrogenic, a sequel of the SAVR. However, the aforementioned findings, although significant, do not provide sufficient evidence to completely exclude the possibility of endocarditis in our patient that may have taken place within the past 10 months. Also, our patient did not have an intraoperative echocardiography report from his previous hospitalization. Thus, there is an ambiguity regarding the primary cause of the patients MLP. Was this a complication of the SAVR or has happened due to other etiologies, such as endocarditis, after the operation? This underlines the importance of performing intraoperative TEE echocardiography in AVR patients.

## Conclusion

In this paper, we discussed MLP, which can occur due to various reasons, such as iatrogenic damage, endocarditis, and autoimmune diseases. During AVR procedure, an intraoperative evaluation with TEE could be beneficial for identifying and treating such condition. We also reported a case of severe MR caused by anterior MLP following AVR which highlights the significance of echocardiography in providing real-time guidance during surgery and detecting the etiology of potential complications.

## Data Availability

The data are available with the corresponding author and can be reached on request.

## References

[1] Witberg G, Codner P, Landes U, Schwartzenberg S, Barbanti M, Valvo R, De Backer O, Ooms JF, Islas F, Marroquin L (2021). Effect of transcatheter aortic valve replacement on concomitant mitral regurgitation and its impact on mortality. Cardiovascular Interventions.

[2] Cortés C, Amat-Santos IJ, Nombela-Franco L, Muñoz-Garcia AJ, Gutiérrez-Ibanes E, De La Torre Hernandez JM, Córdoba-Soriano JG, Jimenez-Quevedo P, Hernández-García JM, Gonzalez-Mansilla A: Mitral regurgitation after transcatheter aortic valve replacement: prognosis, imaging predictors, and potential management. *JACC: Cardiovascular Interventions* 2016, 9:1603–1614. 10.1016/j.jcin.2016.05.025 27491611

[3] Kumar N, Kumar JE, Hussain N, Gorelik L, Essandoh MK, Whitson BA, Bhatt AM, Flores AS, Hachem A, Sawyer TR: New or worsened mitral regurgitation after surgical aortic valve replacement: a systematic review. In *Proceedings of the Seminars in Cardiothoracic and Vascular Anesthesia2021*. SAGE Publications Sage CA: Los Angeles, CA:173–184. 10.1177/1089253220982202 33356967

[4] Essandoh M, Otey A, Bhandary S, Crestanello J (2015). Severe mitral regurgitation complicating minimally invasive aortic valve replacement: Is it functional or organic?. J Cardiothorac Vasc Anesth.

[5] Barreiro CJ, Patel ND, Fitton TP, Williams JA, Bonde PN, Chan V, Alejo DE, Gott VL, Baumgartner WA: Aortic valve replacement and concomitant mitral valve regurgitation in elderly individuals: impact on survival and functional outcome. *Circulation* 2005, 112:I-443-I-447. 10.1161/CIRCULATIONAHA.104.526046 16159860

[6] Reda Abuelatta M, Hesham Naeim M, Ahmad AlAhmadi M (2018). Saleh Al Ghamdi M, Osama Amoudi M, Ibraheem AlHarbi M, Abdelfatah Elasfar M: Transcatheter repair of anterior mitral leaflet perforation in a patient with mechanical aortic valve using antegrade and retrograde approaches: case report. Journal of Structural Heart Disease.

[7] Sazzad F, Xian OZ, Ler A, Guohao C, Swee KG, Kofidis T (2021). Incidence of valvular regurgitation and leaflet perforation by using automated titanium fasteners (CORKNOT®) in heart valve repair or replacement: less usual than reported. J Cardiothorac Surg.

[8] Van Dyck M, Glineur D, de Kerchove L, El Khoury G (2013). Complications after aortic valve repair and valve-sparing procedures. Annals of cardiothoracic surgery.

[9] Hahn RT (2014). Guidance of transcatheter aortic valve replacement by echocardiography. Curr Cardiol Rep.

[10] Veronesi F, Corsi C, Sugeng L, Mor-Avi V, Caiani EG, Weinert L, Lamberti C, Lang RM: A study of functional anatomy of aortic-mitral valve coupling using 3D matrix transesophageal echocardiography. *Circulation: Cardiovascular Imaging* 2009, 2:24–31. 10.1161/CIRCIMAGING.108.785907 19808561

[11] Lin BA, Forouhar AS, Pahlevan NM, Anastassiou CA, Grayburn PA, Thomas JD, Gharib M (2010). Color Doppler jet area overestimates regurgitant volume when multiple jets are present. J Am Soc Echocardiogr.

[12] Zoghbi WA, Enriquez-Sarano M, Foster E, Grayburn PA, Kraft CD, Levine RA, Nihoyannopoulos P, Otto CM, Quinones MA, Rakowski H (2003). Recommendations for evaluation of the severity of native valvular regurgitation with two-dimensional and Doppler echocardiography. J Am Soc Echocardiogr.

[13] Bach DS: Echo/Doppler evaluation of hemodynamics after aortic valve replacement: principles of interrogation and evaluation of high gradients. *JACC: Cardiovascular Imaging* 2010, 3:296–304. 10.1016/j.jcmg.2009.11.009 20223428

